# Remote Telesurgery Versus Local Minimally Invasive Surgery: A Systematic Review of the Comparative Evidence Base

**DOI:** 10.1002/rcs.70224

**Published:** 2026-08-15

**Authors:** Sara Ben Hmido, Houssam Abder Rahim, Faridi S van Etten‐Jamaludin, Dirk Ubbink, Frank Bloemers

**Affiliations:** ^1^ Department of Surgery Amsterdam University Medical Center Location Free University Amsterdam the Netherlands; ^2^ Cancer Center Amsterdam Amsterdam the Netherlands; ^3^ Biomedical Engineering Technical University Delft Delft the Netherlands; ^4^ Research Support Medical Library Amsterdam University Medical Center Location Academic Medical Center Amsterdam the Netherlands; ^5^ Department of Surgery Amsterdam University Medical Center Location Academic Medical Center Amsterdam the Netherlands; ^6^ Department of Trauma Surgery Amsterdam University Medical Center Location Academic Medical Center Amsterdam the Netherlands

**Keywords:** efficacy, efficiency, minimally invasive, surgery, systematic review, telesurgery

## Abstract

**Background:**

Remote telesurgery allows surgeons to operate over a distance using robotic systems and advanced networks. This review aims to compare remote telesurgery with local minimally invasive surgery (MIS) regarding patient outcomes and system‐level outcomes.

**Methods:**

A PRISMA‐compliant systematic review (PROSPERO CRD42023474196) searched six databases to 1 August 2025 for studies comparing remote telesurgery with local MIS in living patients.

**Results:**

Among 5737 records, one retrospective cohort met the criteria. It compared 5G‐enabled remote robotic pelvic fracture surgery (*n* = 80) with local robotic surgery (*n* = 80). Remote procedures had lower blood loss (20 vs. 50 mL) and shorter incisions (1.25 vs. 1.5 cm). Operative time did not significantly differ between groups (*p* = 0.177), and complications, hospital stay, and functional outcomes were similar.

**Conclusions:**

Comparative data on telesurgery remain limited, and healthcare system benefits are unproven. Future studies should incorporate direct comparisons and systematically evaluate system‐level outcomes.

## 1 Introduction

Over the past three decades, minimally invasive surgery (MIS) has become the preferred approach for many surgical procedures, offering reduced postoperative pain, reduced surgical site infection, shorter hospital stays, and faster return to normal activity compared with traditional open surgery [1]. Coupled with advances in imaging, instrumentation, and surgical robotics, MIS has continually evolved to expand the boundaries of what is technically and clinically possible [2]. One of the most ambitious developments in this trajectory is *remote telesurgery*, a technique in which the surgeon operates from a location geographically distant from the patient, using robotic systems and high‐speed telecommunication networks to perform the procedure in real time [3].

The concept of remote surgery captured public imagination in 2001, when the first transatlantic robotic cholecystectomy was successfully performed, marking a technological milestone [4]. Since then, proof‐of‐concept procedures have been documented in various settings, often accompanied by considerable lay press attention [5, 6, 7]. Academic case reports have since extended this concept across other surgical specialties, including urology, hepatobiliary and thoracic surgery, and thyroid surgery, using 5G and other high‐speed networks to connect surgical centres separated by tens to thousands of kilometres [8, 9, 10, 11].

The promise of remote surgery is compelling: expert surgeons could operate on patients in underserved, rural, or geographically isolated areas without the need for physical travel or with peer education purposes, potentially reducing delays in treatment and improving equity in surgical care access. However, the real‐world implementation of this approach depends on several prerequisites, including reliable high‐speed connectivity, access to affordable robotic platforms, and the availability of trained local healthcare teams to support procedures and manage potential complications [12].

Despite technical feasibility and scattered reports of clinical success, the actual efficacy of remote telesurgery relative to local MIS remains unclear. Much of the literature to date has focused on technical descriptions, system feasibility, or case series and reports [13]. While these demonstrate that the technology works, they provide limited evidence on key questions that matter to patients and health systems alike: whether patient outcomes are equivalent or superior when surgery is performed remotely, and how this approach affects healthcare system efficiency, logistics, costs, and safety.

Given the increasing global surgical volume, persistent disparities in access to specialist surgeons, and the investments required for telesurgical infrastructure, it is essential to rigorously evaluate these questions [14]. Without such evidence, adoption decisions around telesurgery risk being driven more by novelty and publicity than by patient benefit and system sustainability.

This systematic review aims to compare the efficacy of remote telesurgery versus local MIS in terms of patient‐related outcomes and healthcare system efficiency among patients requiring surgical procedures.

## 2 Materials and Methods

A literature review was conducted following PRISMA guidelines [15]. The systematic literature search protocol was registered in the PROSPERO database under the number CRD42023474196 [16].

### 2.1 Definitions

Telesurgery was defined as the performance of a surgical procedure by a surgeon who is physically distant from the patient, beyond direct line of sight and separated by a substantial geographic distance such as being in a different building, city, or country rather than in another room of the same facility. The procedure was to be conducted using computer‐assisted robotic systems to manipulate instruments in real time, with the surgeon's commands and sensory feedback, including visual or haptic information, transmitted bidirectionally over a telecommunications network. In this context, the term applies only to procedures performed on live patients.

### 2.2 Literature Search

A literature search was conducted, with the help of a clinical librarian (FvEJ), in Medline (PubMed), Embase (Ovid), Cochrane Library, Web of Science, Scopus, and IEEE Xplore to identify studies comparing remote telesurgery with minimally invasive in‐person surgery. The databases were searched from their inception until 1 August 2025.

Searches in Medline and Embase combined controlled vocabulary (MeSH and Emtree terms), entry terms, and free‐text terms. Web of Science, Scopus, and IEEE Xplore were searched using free‐text terms only, while the Cochrane Library employed a combination of controlled vocabulary and free‐text terms.

The search strategy was built around two concept blocks. The first block included terms relating to remote telesurgery such as ‘remote surgery’, ‘telesurgery’, ‘tele surgery’, ‘telerobotic surgery’, ‘tele robotic surgery’, ‘remote robotic surgery’, ‘cyber‐surgery’, ‘remote surgical robot*’, ‘remote surgical procedure*’, and ‘remote surgical resection*’. The second block included terms relating to minimally invasive and robotic surgery such as ‘keyhole surgery’, ‘less invasive surgery’, ‘minimally invasive surgery’, ‘minimal invasive surgery’, ‘mini‐invasive surgery’, ‘endoscopy’, ‘laparoscopy’, and ‘robot*’. These blocks were combined with Boolean operators to ensure comprehensive retrieval of relevant studies.

Detailed search syntaxes for each database are provided in Supporting Information S2.

### 2.3 Study Selection

#### 2.3.1 Inclusion and Exclusion Criteria

Studies were included in this systematic review if they met the following criteria: (1) the surgical procedure was completed remotely with the surgeon physically separated from the patient by a substantial geographic distance between the console and patient‐side platform, (2) the procedure was performed on living patients, and (3) remote surgery was compared to a traditional (non‐remote) minimally invasive surgical approach within the same study. No language restrictions were applied. References in eligible articles were screened for other relevant publications. No trial registers were searched for ongoing studies.

Studies were excluded if they (1) did not involve living human subjects (e.g., cadaveric, animal, or simulator studies), (2) examined procedures where the surgeon and the robotic platform were located in the same room or facility without meaningful geographic separation, (3) focused solely on telementoring without direct instrument control by the remote surgeon, or (4) lacked a comparator intervention such as in case reports or non‐comparative case series.

#### 2.3.2 Screening

Upon eliminating duplicate entries, the screening process was divided into two phases: the initial phase involved an examination of titles and abstracts, followed by a second phase that entailed an analysis of the full‐text articles to confirm the selection of studies. Articles published in languages other than English or Dutch were translated using the document translation function in Google Translate.

Two authors (S.B., H.A.) independently performed both the title/abstract screening and full‐text analysis to ensure consistency and accuracy. Any discrepancies were assessed by a third reviewer (D.U.). No additional articles were identified through the snowball search.

#### 2.3.3 Critical Appraisal

The risk of bias in included studies was assessed using the Risk of Bias in Non‐Randomized Studies‐of interventions (ROBINS‐I) methodology, considering the criteria used in each of its seven domains: bias due to confounding, bias in the selection of participants, bias in the classification of interventions, bias due to missing data, measurement of outcomes, and bias in the selection of reported results.

#### 2.3.4 Effect Measures and Synthesis

Median differences and odd ratios with 95% confidence intervals were used as effect measures when reported in the included study. As only one comparative study met the inclusion criteria, no quantitative synthesis or meta‐analysis was performed.

#### 2.3.5 Ethics Statement

This systematic review used only publicly available data from published studies and required no ethical approval.

## 3 Results

**FIGURE 1:**
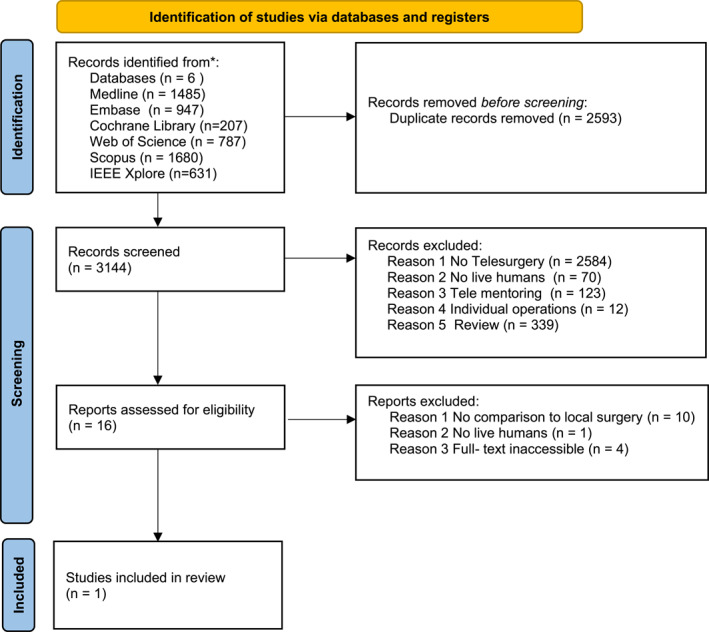
PRISMA flow diagram of study selection. One study met the inclusion criteria.

The search strategy yielded a total of *n* = 5737 records. An overview of the study selection process, the following PRISMA guidelines, is presented in Figure 1.

Notably, one article randomised and compared telesurgery with local surgery, but this was performed between surgeons using a gallbladder model rather than in living patients [17]. Another study compared two telesurgery cases, but the comparison was between a 1700 km long‐range and a 70 km short‐range procedure. [18].

Ultimately, one article met the inclusion criteria.

### 3.1 Study Characteristics

The sole included study was a retrospective cohort comparing 5G remote robotic‐assisted surgery with local robotic‐assisted surgery for pelvic fractures. The cited reason for employing telesurgery was the uneven distribution of high‐quality medical resources, leaving smaller and regional hospitals with limited capacity and restricting access to quality care for pelvic fracture patients [19].

The two centres were located 2161 km apart: Beijing Jishuitan Hospital, which provided remote expert guidance, and Foshan Hospital of Traditional Chinese Medicine, where the surgeries were performed. A total of 160 patients were included, 80 in each group. All patients underwent percutaneous screw fixation for pelvic fractures using the TiRobot orthopaedic surgical robot, either with remote 5G guidance or locally performed robotic assistance [19]. Baseline characteristics, including sex, age, BMI, injury mechanism, and fracture classification, were similar between groups (see Table 1).

**TABLE 1:** Baseline characteristics and outcome indicators (*n* = 80 per group) of Dai et al. [19].

Variable	5G group	Control group	Effect size (95% CI)	*p* value
Comparison of baseline data				
Sex (male/female)	39/41	52/28	—	0.055
Age (years, mean ± SD)	48.74 ± 18.74	50.14 ± 17.36	—	0.625
Body mass index (kg/m^2^, mean ± SD)	22.70 ± 3.14	22.75 ± 3.09	—	0.912
Disease duration (days, median [IQR])	10.0 (7.25–12.75)	10.0 (7.0–16.0)	—	0.164
Cause of injury (fall/high fall/traffic accident/crush/strike)	15/15/41/9/0	12/24/35/8/1	—	0.41
Fracture classification (tile A/B/C)	2/44/34	1/38/41	—	0.852
Comparison of outcome				
Operative time (min, median [IQR])	99.0 (65.3–147.3)	120.0 (67.3–169.0)	MD −13.0 (−33.0 to 5.0)	0.177
Intraoperative blood loss (mL, median [IQR])	20.0 (10.0–50.0)	50.0 (20.0–100.0)	MD −20.0 (−40.0 to 0.0)	0.001
Incision length (cm, median [IQR])	1.25 (1.0–3.0)	1.5 (1.5–3.0)	MD 0.0 (−0.5 to 0.0)	0.025
Hospital stay (days, median [IQR])	16.0 (11.3–24.0)	18.5 (12.0–29.0)	MD −2.0 (−5.0 to 1.0)	0.206
Maximum residual displacement (mm, mean ± SD)	3.10 ± 5.48	3.85 ± 7.15	MD 0.0 (−1.1 to 0.0)	0.407
Reduction quality (excellent/good/fair/poor)	52/27/1/0	46/32/2/0	—	0.557
Screw placement accuracy (excellent/good/poor)	172/5/3	195/9/9	—	0.243
Follow‐up time (months, median [IQR])	11.5 (9.25–13.0)	12.0 (9.0–13.0)	MD 0.0 (−1.0 to 1.0)	0.78
Majeed pelvic function score (median [IQR])	95.0 (85.5–98.0)	95.0 (80.0–100.0)	MD 0.0 (−3.0 to 1.0)	0.687
Majeed function grading (excellent/good/fair/poor)	62/15/2/1	53/24/2/1	—	0.375
Gait alteration (no limp/mild/moderate/severe)	74/3/0/3	65/11/2/2	—	0.041
Squatting limitation, *n* (%)	1 (1.25%)	2 (2.5%)	OR 2.03 (0.18 to 22.80)	1
Persistent pain, *n* (%)	4 (5.0%)	5 (6.25%)	OR 1.27 (0.33 to 4.90)	1

In the 5G group, the connection was established using a 5G CPE router (customer premises equipment, providing 5G access in the operating room) linked to the TiRobot surgical platform. The operating room joined the remote session via an MCU address with login credentials. Three PTZ (pan‐tilt‐zoom) cameras provided remotely adjustable high‐definition video feeds, while wireless headsets enabled real‐time audio communication. Imaging from C‐arm fluoroscopy and the robotic planning interface were transmitted over the 5G network to the remote team. Remote experts planned screw trajectories and positioned the robotic arm, while local surgeons inserted guide pins, placed screws, and closed incisions.

### 3.2 Study Results

The authors noted that the 5G connection remained stable throughout, without failures, delays, or technical issues. Although they referenced theoretical 5G performance, no latency measurements were reported.

Key perioperative outcomes are also presented in Table 1. Intraoperative blood loss was significantly lower in the 5G group (median 20 mL) compared to the control group (median 50 mL, *p* = 0.001). Incision length was also shorter in the 5G group (median 1.25 vs. 1.5 cm, *p* = 0.025). A total of 180 screws were implanted in the 5G group compared with 213 in the control group. Dai et al. suggested this reflected a more efficient trajectory planning by the remote expert team. Operative time tended to be shorter in the 5G group (median 99 vs. 120 min), but this difference was not statistically significant (*p* = 0.177). Hospital stay was similar between the groups. Functional outcomes were comparable, with both groups achieving a median Majeed pelvic function score of 95 at a median follow‐up of 11.5 months (IQR 9.25–13.0) in the 5G group and 12.0 months (IQR 9.0–13.0) in the control group, although gait alterations were reported less frequently in the 5G group (*p* = 0.041) [19].

No healthcare system–level metrics, such as cost, workforce efficiency, or resource use, were reported. Potential system benefits, such as improved access to expert care in underserved regions, were discussed conceptually but without empirical data.

### 3.3 Critical Appraisal

The study overall showed a moderate risk of bias (see Table 2).

**TABLE 2:** Quality assessment of the included studies.

Study	Bias due to confounding	Bias in selection of participants into the study	Bias in classification of interventions	Bias due to deviations from intended interventions	Bias due to missing data	Bias in measurement of outcomes	Bias in selection of the reported results
Dai Yonghong (2025)							

*Note:* Green stands for low risk of bias, Red stands for high risk of bias, Yellow stands for unclear risk of bias.

As with any retrospective design, this study is inherently susceptible to selection bias, since patients were not randomised to remote or local surgery. How patients were assigned to each group is not reported, so this domain was rated as unclear risk rather than low risk.

There was no indication of bias in the classification of interventions or in deviations from the intended interventions, as both groups underwent the same core surgical procedure with only the location of surgical planning differing between arms.

There is also a potential for bias due to confounding, as the retrospective design may allow residual confounding despite comparable baseline characteristics between groups. Notably, the 5G group involved joint input from an additional expert surgical team at Beijing Jishuitan Hospital, whereas the control group was managed solely by the local Foshan team; this difference in surgical expertise, rather than the remote technology itself, is a plausible unmeasured confounder that baseline characteristics alone cannot rule out.

Additionally, the assessment of outcomes, such as the Majeed score, was not performed blindedly, and this domain was rated as high risk given the potential for unblinded assessors to be influenced by knowledge of group allocation. Given the lack of randomisation, unclear allocation process, and unblinded outcome assessment, the reliability of the results should be considered moderate.

## 4 Discussion

This literature review yielded some comparative evidence on the effectiveness of remote telesurgery against local minimally invasive surgery, in terms of patient outcomes and healthcare system efficiency. Despite an extensive search, only one comparative article met the inclusion criteria.

The single included study, comparing 5G remote robotic‐assisted pelvic fracture surgery with local robotic‐assisted surgery, reported similar operative success, complication rates, and functional outcomes between groups, and even suggested lower blood loss and smaller incisions in the remote group. This finding may suggest a potential functional advantage of the telesurgical approach; however, its interpretation remains uncertain given the retrospective design and the lack of detailed reporting on baseline mobility, assessment methods, and potential confounding factors. Whether this difference reflects a true surgical benefit or methodological bias requires confirmation in prospective comparative studies.

Although statistically significant, the presented differences were small and their clinical relevance remains uncertain, as both procedures resulted in minimal blood loss and similar clinical outcomes. In addition, the control group received a higher total number of screws than the remote group. This may represent a potential confounding factor, as greater fixation requirements could have contributed to longer operative times and larger incisions independent of the telesurgical approach.

Importantly, the study did not provide measurements of latency or any healthcare system–level metrics such as cost, resource use, or efficiency. The absence of these data limits the extent to which its findings can inform broader adoption or policy decisions. Furthermore, although the study described the connection as stable without delays or technical failures, this assessment was based on qualitative reporting rather than objective measurements of network performance. The absence of latency data makes it difficult to independently verify these stability claims or determine whether the reported performance is reproducible across different clinical settings and network conditions.

It is also worth noting that the included study reflects a hybrid telesurgical model rather than a fully remote procedure: remote experts planned the screw trajectory and positioned the robotic arm, while local surgeons performed the tactile‐sensitive steps of guide pin insertion, screw placement, and incision closure. This division of labour is relevant to interpreting the findings, since retaining tactile‐sensitive steps on‐site likely explains why haptic feedback transmission, a recognised technical challenge in remote telesurgery, was not reported. The results should therefore be read as evidence for this specific hybrid model, rather than for fully remote procedures in which the remote surgeon performs tactile‐sensitive manoeuvres directly.

Compared with the 2022 review by Barba et al., our 2025 review finds 10 additional case studies and 9 case series, and the only included comparative article nearly all from China, showing that most recent telesurgery development has occurred there [8, 9, 10, 11, 13, 18, 19, 20, 21, 22, 23, 24, 25, 26, 27, 28, 29, 30, 31, 32, 33].

These reports span a range of surgical specialties, including urology [8, 21, 22, 24, 25, 26, 30, 31, 32], hepatobiliary and pancreatic surgery [9, 23, 27], thoracic surgery [10, 28], and gynaecological and head‐and‐neck procedures [11, 29], most performed using 5G connectivity across distances ranging from tens to over 2000 km, with several explicitly framed as ultra‐remote or transnational cases [28, 29, 32]. Taken together, these studies demonstrate that telesurgery is technically feasible and can be performed in live patients.

The consistent completion of reported operations without conversion, together with qualitative reports of stable network performance, suggests that modern telecommunications and robotic platforms can support telesurgical procedures in controlled environments. However, these findings should be interpreted cautiously given the uncontrolled nature of available studies, lack of objective latency measurements, and potential publication bias.

Wat remains absent is evidence that telesurgery delivers meaningful benefits at the healthcare system level. Metrics such as cost, efficiency, resource utilisation, and workforce implications are either missing or only described anecdotally. Although formal economic evaluations were not available, the included comparative study illustrates that telesurgical implementation requires substantial technological infrastructure, including dedicated 5G communication equipment, PTZ cameras, and supporting network systems. These setup requirements may represent important cost considerations that should be evaluated in future health‐economic analyses.

Without these data, it is difficult to determine whether telesurgery will ultimately enhance system sustainability, reduce disparities, or instead introduce new logistical, financial, or expert workforce challenges. Beyond clinical and operational considerations, broader adoption of telesurgery will also depend on regulatory frameworks and liability structures. Differences in licencing requirements, cross‐border practice regulations, data governance, and responsibility in the event of adverse outcomes remain important unresolved issues across jurisdictions.

Future research should move beyond proof‐of‐concept case series that primarily demonstrate technical feasibility. Greater value can come from comparative study designs that explicitly evaluate telesurgery against locally performed minimally invasive surgery. Importantly, reporting should extend beyond clinical outcomes to encompass system‐level metrics such as procedure cost, infrastructure requirements, training needs, and the efficiency of surgical workforce deployment. Collecting such data would provide the robust evidence needed to guide the responsible clinical integration of telesurgery.

### 4.1 Limitations

The resulting evidence is limited by the scarcity of comparative studies, with only a single eligible article identified. The additional case series, while valuable in demonstrating technical feasibility, does not provide comparative or system‐level data, restricting the conclusions that can be drawn. This scarcity is compounded by the geographic concentration of the available evidence, almost entirely from China, which introduces a risk of bias and limits generalisability: differences between countries in telecommunications infrastructure, regulatory oversight, robotic platform availability, and reimbursement structures mean the outcomes reported here may not transfer directly to other health systems.

Several included and excluded studies were published in Chinese, and although machine translation using Google Translate enabled their assessment, this approach carries a risk of misinterpreting clinical and technical details. The absence of a native Chinese‐speaking reviewer may have further limited the ability to capture language‐specific nuances.

The evidence base is also constrained by what was, and was not, measured. Publication bias may have shaped what is available, as unsuccessful or aborted telesurgical procedures are less likely to be reported. Objective assessments of technical performance, including latency measurements and network reliability, were rarely provided, limiting the ability to independently evaluate the reliability of current telesurgical platforms. The included study likewise did not report a sample size or power calculation, raising the possibility that it was underpowered to detect differences in outcomes such as operative time.

Finally, the included study reflects a hybrid division of labour rather than a fully remote procedure, and provides no data on haptic feedback transmission; its findings may not generalise to fully remote procedures in which the remote surgeon performs tactile‐sensitive manoeuvres directly.

### 4.2 Conclusion

Telesurgery is technically feasible and holds the promise of expanding access to expert surgical care in underserved regions. However, comparative evidence is scarce, and healthcare system benefits remain speculative. The field would be greatly advanced if future studies incorporated direct comparisons and systematically reported on system‐level outcomes, including cost and resource use, in addition to patient outcomes.

## Funding

The authors have nothing to report.

## Conflicts of Interest

The authors declare no conflicts of interest.

## Supporting information


Supporting Information S1

Supporting Information S2

## Data Availability

All data analysed in this review are available within the published article by Dai et al. (2025) and its supplementary materials. The search strategies for all six databases are provided in Supporting Information S2: Tables S1–S6. No new data were generated for this review.
