# Supplementary material for: Remote Telesurgery Versus Local Minimally Invasive Surgery: A Systematic Review of the Comparative Evidence Base
Source: Int J Med Robot. 2026 Aug 15;22(4):e70224. doi: 10.1002/rcs.70224 (PMC13477495; doi:10.1002/rcs.70224)
Supplement: Supplementary file 2 [file RCS-22-e70224-s002.docx]

| **Table S1**  Search strategy: Medline | |
| --- | --- |
| Search | PubMed query- August 1 |
| #1 | ("Telemedicine"[Mesh] AND ("Surgical Procedures, Operative"[Mesh] OR "General Surgery"[Mesh]) OR remote surger*[tiab] OR telesurg*[tiab] OR tele surg*[tiab] OR telerobotic surg*[tiab] OR tele robotic surg*[tiab] OR remote robotic surg*[tiab] OR cyber-surg*[tiab] OR remote surgical robot*[tiab] OR remote surgical proced*[tiab] OR remote surgical resec*[tiab]) AND ("Minimally Invasive Surgical Procedures"[Mesh] OR "Laparoscopy"[Mesh] OR "Endoscopy"[Mesh] OR "Robotic Surgical Procedures"[Mesh] OR "Robotics"[Mesh] OR keyhole surgery[tiab] OR less invasive surgery[tiab] OR minimally invasiv*[tiab] OR minimal invasiv*[tiab] OR mini-invasiv*[tiab] OR endoscop*[tiab] OR laparoscop*[tiab] OR robot*[tiab]) |

| **Table S2**  Search strategy: Embase | | |
| --- | --- | --- |
| Search | | Embase  query- August 1 |
|  | #1 | telesurgery/ |
|  | #2 | (remote surger* or telesurg* or tele surg* or telerobotic surg* or tele robotic surg* or remote robotic surg* or cyber-surg* or remote surgical robot* or remote surgical proced* or remote surgical resec*).ti,ab,kf. |
|  | #3 | 1 or 2 |
|  | #4 | minimally invasive surgery/ or exp laparoscopy/ or endoscopy/ or exp robot assisted surgery/ or exp robotics/ |
|  | #5 | (keyhole surgery or less invasive surgery or minimally invasiv* or minimal invasiv* or mini-invasiv* or endoscop* or laparoscop* or robot*).ti,ab,kf. |
|  | #6 | 4 or 5 |
|  | #7 | 3 and 6 |

**Table S3**

Search strategy: Cochrane Library

| Search | Cochrane Library  query-August 1 |
| --- | --- |
| #1 | (remote surger* or telesurg* or tele surg* or telerobotic surg* or tele robotic surg* or remote robotic surg* or cyber-surg* or remote surgical robot* or remote surgical proced* or remote surgical resec*):ti,ab,kw |
| #2 | (keyhole surgery or less invasive surgery or minimally invasiv* or minimal invasiv* or mini-invasiv* or endoscop* or laparoscop* or robot*):ti,ab,kw |
| #3 | #1 and #2 in Cochrane Reviews, Trials |

**Table S4**

Search strategy: Web of Science

| Search | Web of Science  query-August 1 |
| --- | --- |
| #1 | TS=("remote surger*" or telesurg* or "tele surg*" or "telerobotic surg*" or "tele robotic surg*" or "remote robotic surg*" or "cyber-surg*" or "remote surgical robot*" or "remote surgical proced*" or "remote surgical resec*") AND TS=("keyhole surgery" or "less invasive surgery" or "minimally invasiv*" or "minimal invasiv*" or "mini-invasiv*" or endoscop* or laparoscop* or robot*) |

**Table S5**

Search strategy: SCOPUS

| Search | SCOPUS   query-August 1 |
| --- | --- |
| #1 | ( TITLE-ABS-KEY ( "remote surger*" OR telesurg* OR "tele surg*" OR "telerobotic surg*" OR "tele robotic surg*" OR "remote robotic surg*" OR "cyber-surg*" OR "remote surgical robot*" OR "remote surgical proced*" OR "remote surgical resec*" ) ) AND ( TITLE-ABS-KEY ( "keyhole surgery" OR "less invasive surgery" OR "minimally invasiv*" OR "minimal invasiv*" OR "mini-invasiv*" OR endoscop* OR laparoscop* OR robot* ) ) |

**Table S6**

Search strategy: IEEE Explore

| Search | IEEE Explore   query-August 1 |
| --- | --- |
| #1 | "remote surgery" OR telesurger* OR "tele surger*" OR "telerobotic surg*" OR "tele robotic surger*" OR "remote robotic surger*" OR "cyber-surgery" OR "remote surgical robot*" OR "remote surgical procedure*" OR "remote surgical resection*" AND "keyhole surgery" OR "less invasive surgery" OR "minimally invasive" OR "minimal invasive" OR "mini-invasive" OR endoscopy OR laparoscopy OR robot* |
